# Cancer Immunotherapy Using AIRE Conditioning of the Tumor Epitopeome

**DOI:** 10.21203/rs.3.rs-5411393/v1

**Published:** 2024-11-15

**Authors:** Richard Vile, Jose Pulido, Alex Chen, Benjamin Kendall, Jason Tonne, Muriel Metko, Jill Thompson, Thanich Sangsuwannukul, Maria Chiriboga Yerovi, Rosa Diaz, Mason Webb, Amanda Huff, Madelyn Moore, Matthew Schuelke, Sheeba Irshad, Elizabeth Appleton, Alan Melcher

**Affiliations:** Mayo Clinic; Mayo Clinic; Mayo Clinic; Mayo Clinic; Mayo Clinic; Mayo Clinic; Mayo Clinic; Mayo Clinic; Mayo Clinic; Mayo Clinic; Mayo Clinic; Johns Hopkins University; Mayo Clinic; Mayo Clinic; King’s College London; The Institute of Cancer Research; Insitute of Cancer Research

## Abstract

T cell immune tolerance is established in part through the activity of the Auto-immune Regulator (AIRE) transcription factor in the medullary Thymic Epithelial Cells (mTEC) of the thymus. AIRE induces expression of SELF peripheral tissue-specific antigens for presentation to naïve T cells to promote activation/deletion of potentially autoreactive T cells. We show, for the first time to our knowledge, that tumors mimic the role of AIRE in mTEC to evade immune rejection. Thus, by expressing a broad range of SELF epitopes against which minimal functional T cell reactivities exist because of thymic deletion, AIRE acts as a master controller of SELFNESS, effectively cloaking the tumor from T cell attack. Moreover, we describe a completely novel immunotherapy in which engineered changes in AIRE expression in tumor cells alters their profile of SELFNESS, exposing both AIRE-modified, and parental unmodified, tumor cells to T cell attack. Consistent with our studies, patient RNAseq shows expression of AIRE predicts response to immune therapies with a strong correlation between AIRE expression and markers of TCR signaling. Therefore, by re-setting the immunological SELFNESS of cancer cells, this novel AIRE-mediated immunotherapy 1). converts a highly tolerized T cell compartment into a heteroclitic tumor-reactive T cell population; 2) confers *de novo* sensitivity to immune checkpoint blockade upon non-immunogenic tumors; 3). completely removes the need to identify potentially immunogenic tumor-associated antigens as targets for generation of *de novo* CD8^+^ and helper CD4^+^ T cell responses; and 4) leads to potent T cell-mediated rejection of aggressive, immunologically cold, non-immunogenic tumors.

## INTRODUCTION

T cell immune tolerance — a state of unresponsiveness towards a specific antigen — is established, and maintained, at least in part through the activity of the Auto-immune Regulator (AIRE) in the thymus^1, 2, 3^. AIRE is a transcription factor expressed principally in medullary thymic epithelial cells (mTEC) which induces expression of a wide range of peripheral tissue specific antigens (TSA). Display of these antigens, and their processed epitopes, to T cells whose T cell receptors (TCR) have high affinity/avidity for the epitopes presented through Major Histocompatibility (MHC) molecules on the mTEC either drives negative selection of these potentially autoreactive T cells (recessive, deletional self-tolerance)^4, 5^ or pushes them into a regulatory T cell (Treg)-dominant lineage (suppressive self-tolerance)^6, 7, 8^. Perturbations of the mechanisms leading to immune tolerance against self-antigens can lead to autoimmunity^2, 9, 10, 11,12, 13, 14^ allergy and hypersensitivity diseases^15^.

When patients present to the clinic with actively growing tumours, the tumour may express only self-antigens to which the immune system is completely unresponsive due to deletion of any potentially tumour reactive T cells. Alternatively, tumours may grow under conditions of functional tolerance in which anti-tumour immune cells exist, recognizing incompletely tolerized self-antigens, neo-antigens or even viral antigens, but are non-functional because they cannot access tumours^16, 17^, are suppressed by immune suppressive tumour microenvironments^18, 19, 20, 21^, become exhausted or anergic^22, 23, 24^, or cannot recognize the tumours due to escape mechanisms such as loss of antigen presenting machinery^25, 26, 27, 28^. Several different approaches are being tried to relieve the multiple pathways which enforce suppressive functional tolerance by uncloaking the pre-existing anti-tumour immune response — using strategies such as immune checkpoint blockade or vaccination^29, 30, 31^.

One approach to overcome recessive (deletional) immune tolerance is to introduce new T cell specificities^32, 33, 34^. An alternative is to generate novel sets of antigenic epitopes on the cancer cells which activate an otherwise absent repertoire of tumour-reactive T cells. In this scenario, it may be possible to generate new individual heteroclitic epitopes, or clusters of heteroclitic epitopes, expressed on cancer cells which are sufficiently immunogenic to prime new T cell reactivities (recognized by high affinity/avidity TCR) but which can react back against the original epitopes probably with lower affinity/avidity but at levels sufficient to lead to tumour rejection ^35, 36, 37^.

The problem remains as to identifying which epitopes are most likely to be effective immunogens at raising *de novo* heteroclitic T cell responses *in vivo*. In addition, targeting single antigens usually leads to antigen loss and tumour escape^25, 26, 27, 28, 38, 39^. Consistent with this, we have shown that vaccination with multi-epitope cDNA libraries is highly effective against tumour growth compared to vaccination with single antigen approaches^40, 41, 42^. Therefore, we hypothesized that induction of a range of multiple tumour-derived epitopes, which can then be selected for immunogenicity and heterocliticity by the patient’s own immune system without having to identify them *a priori*, could be a successful vaccination approach to treat a variety of tumours. Since expression of AIRE in mTECs is a key driver of the expression of ‘SELFNESS’ for negative selection of autoreactive T cells in the thymus^12^, we hypothesized that AIRE expression in cancer cells^43^ — at high, intermediate or low levels — may mimic the thymic process of negative selection of autoreactive T cells but with the opposite goal of setting a profile of SELFNESS (defined as the profile of epitopes available for a lack of T cell reactivity on tumour cells) which is designed to avoid T cell activation and recognition, thereby contributing directly to the immune (in)visibility of tumours. Thus, on a tumour cell-by-cell basis, a certain level of AIRE might drive the optimal profile of [SELF + Neo-Antigen + Viral/Foreign antigen] epitopes that prevent T cell recognition by excluding display of epitopes to which T cell reactivities exist. If that were true, by changing the profile of SELFNESS away from that specifically selected as conferring low immunogenicity, changing levels of AIRE within tumours may act as a reset mechanism allowing *de novo* priming of novel T cell repertoires against the AIRE modified cancer cells, subsets of which may possess heteroclitic properties which react against the SELF epitopes expressed by the unmodified tumour cells. In this scenario, engineering changes in the levels of AIRE could be used as a master transcriptional switch to alter the SELFNESS of tumour cells to generate T cell reactivities against them.

Whereas some tumour types such as melanoma with high mutational loads are considered to be inherently immunogenic, others, such as the paediatric high-grade glioma Diffuse Midline Glioma (DMG) are generally very poorly immunogenic (low mutational loads), non-immune infiltrated and manifest as aggressive diseases with a two-year median survival of less than 10%^44, 45^. Identification of H3K27M mutations in greater than 85% of midline infiltrating gliomas^46^ globally rewires the cellular transcriptional program in concert with other frequently occurring mutations in *ACVR1*, TP53, ATRX and PDGFRA^47, 48, 49, 50^.

Therefore, here we tested the hypothesis that alterations in AIRE expression can be used to alter the immunogenicity of both immunogenic melanomas as well as poorly immunogenic DMG to render them sensitive to immunotherapy. We show that manipulation of AIRE levels in tumour cells leads to a change in the profile of epitopes presented by MHC Class I molecules and that the altered profile of epitopes significantly alters the SELFNESS of tumour cells as measured by their ability to activate autologous T cell responses. Moreover, we show that this AIRE-induced altered SELFNESS stimulates T cell mediated rejection of parental tumours *in vivo* either through a dendritic cell-mediated vaccination route or by *in vivo* delivery of AIRE-expressing vectors. Overall, our data here are significant in that they demonstrate that altering the epitopeome of cancer cells is a very effective immunotherapeutic strategy to convert a highly tolerized T cell compartment into a heteroclitic tumour reactive T cell population. Thus, changing AIRE levels acts as a master switch to re-engineer tumour SELFNESS without the need to identify any specific antigens which need to be manipulated to stimulate anti-tumour T cell responses.

## RESULTS

### AIRE expression in tumour cells

**Figure 1.**
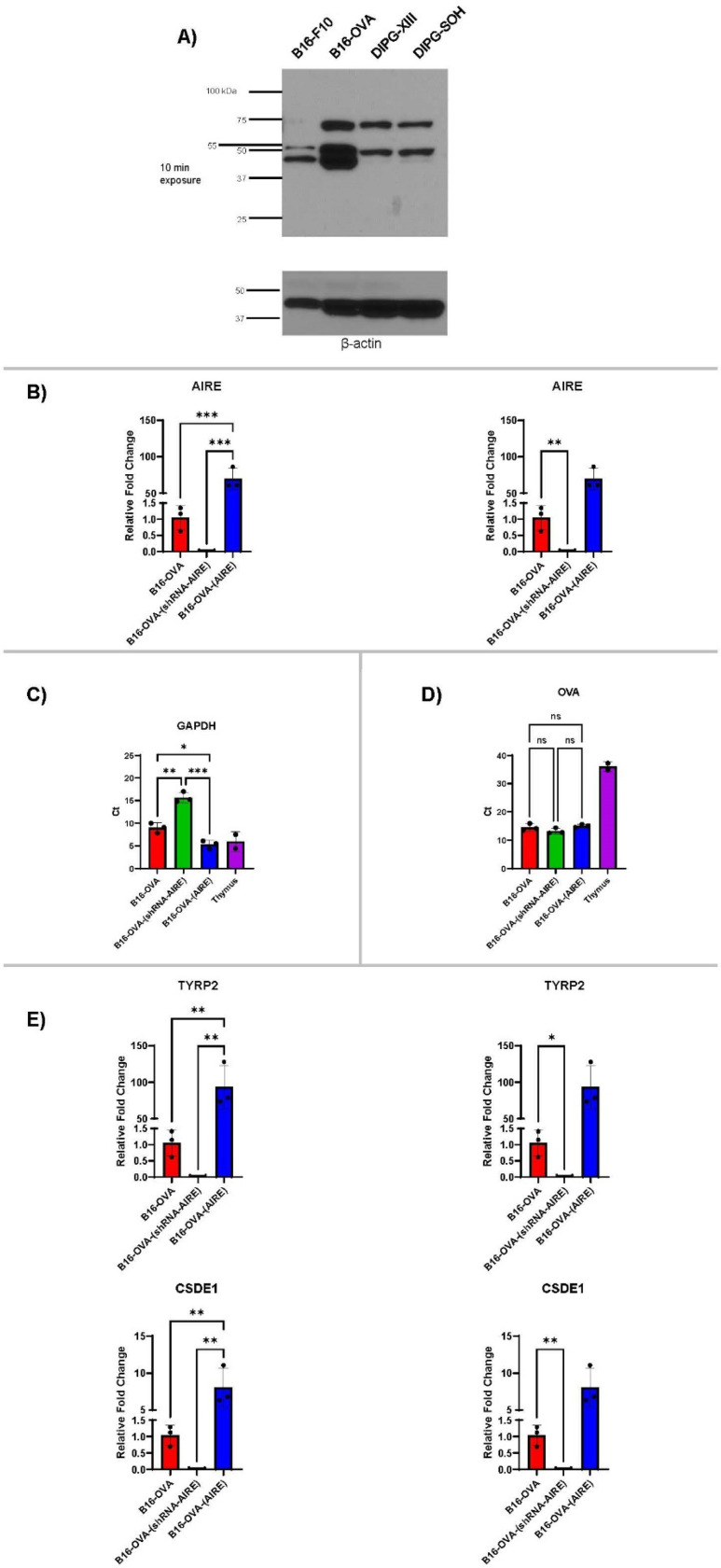
AIRE regulates expression of SELF proteins, but not OVA, in murine B16 tumour cells. **A.** 10^7^ murine melanoma B16-F10 or B16-F1-OYA cells, or human DIPG-XIII or DIPG-SOH Diffuse Midline Glioma cell lines were analyzed by Western Blot for expression of AIRE using the rabbit polyclonal 22517–1AP antibody at 1:500 dilution https://www.ptglab.com/products/AIRE-Antibody-22517-1-AP.htm (Proteintech, Rosemont, IL, USA). **B.** qrtPCR for levels of AIRE expression in B16-OVA, B16-OVA-(shRNA-AIRE) (B16-OVA stably transfected with shRNA against murine AIRE) or B16–0VA-(AIRE) (B16-OVA stably transfected with CMV-murine AIRE expression plasmid) cells. Expression of AIRE in two independent mouse thymi was used as a control. **C.** Mean CT levels for qrtPCR of GAPDH are shown for B16-OVA. B16-OVA-(shRNA-AIRE) and B16–0VA-(AIRE) cells. Mean values represent three biological replicates. CT values of two independent mouse thymx was used as a control. **D.** Mean CT levels for qrtPCR of OVA are shown for B16-OVA, B16-OVA-(shRNA-AIRE) and B16-OVA-(AIRE) cells. Mean values represent three biological replicates. CT values of two independent mouse thymi was used as a control. **E.** qrtPCR for levels of TYRP2 and CSDE1 expression relative to OVA in B16-OVA, B16-OVA-(shRNA-AIRE) and B16–0VA-(AIRE) cells. ΔΔC_t_ method is shown. In both cases, levels of TYRP2 and CSDE1 expression m B 16-OVA vs. B16-OVA-<shRNA-AIRE) were not statistically significant using ANOVA (left hand side) using the 2^−*ΔΔCt*^ method for comparative analysis: • *ΔCt*_*CSDE1*_ = *Ct*_*CSDE1*_ - *Ct*_*OVA*_ • Standardize Ct to endogenous OVA expression • *ΔΔCt*_*CSDE1*_ = *ΔCt*_*CSDE1*_–*ΔCt*_*B16ova-OVA-average*_ • Standardize to B16-OVA control • *fold gene expression* = 2^−ΔΔ*Ct*^ • Relative gene expression of CSDE1 with respect to B16-OVA However, levels of TYRP2 and CSDE1 expression in B16-OVA vs. B16–0VA-(shRNA-AIRE) were both highly significant using an impaired t-test (right hand side).

B16-F10 and B16-F10-OVA murine melanoma cell lines had different patterns of AIRE expression, and two human paediatric Diffuse Midline Glioma cell lines (DIPG-XIII and DIPG-SOH) had similar AIRE profiles (Fig. 1A). Because of the availability of reagents to monitor T cell responses to the immunodominant SIINFEKL CD8^+^ T cell epitope of the OVA protein, we generated B16-OVA cells lines in which AIRE was either knocked down or was over-expressed using stable transfection with a plasmid in which murine AIRE was constitutively expressed by the CMV promoter (Fig. 1B). Knockdown of AIRE significantly reduced levels of GAPDH, whilst over-expression of AIRE also increased GAPDH levels (Fig. 1C). In contrast, levels of OVA RNA were very similar between all three cell lines (Fig. 1D). These data argue that OVA will be a more suitable reference gene for comparison of expression levels of AIRE than GAPDH. We also showed that the levels of two well characterized SELF proteins, TYRP2 and CSDE1, were both significantly decreased in B16-OVA-(shRNA-AIRE) cells and increased in B16-OVA-(AIRE) cells relative to levels of OVA expression (Fig. 1E). Thus, consistent with the reported dependency of SELF melanoma associated antigen expression upon AIRE^51, 52^, GAPDH, TYRP2 and CSDE1, three well-characterized SELF proteins, are regulated by AIRE in B16-OVA cells, but expression of stably transfected, CMV-driven OVA protein was independent of AIRE.

### AIRE-mediated MHC I Occupancy Controls Presentation of Foreign Epitopes and SELFNESS

**Figure 2:**
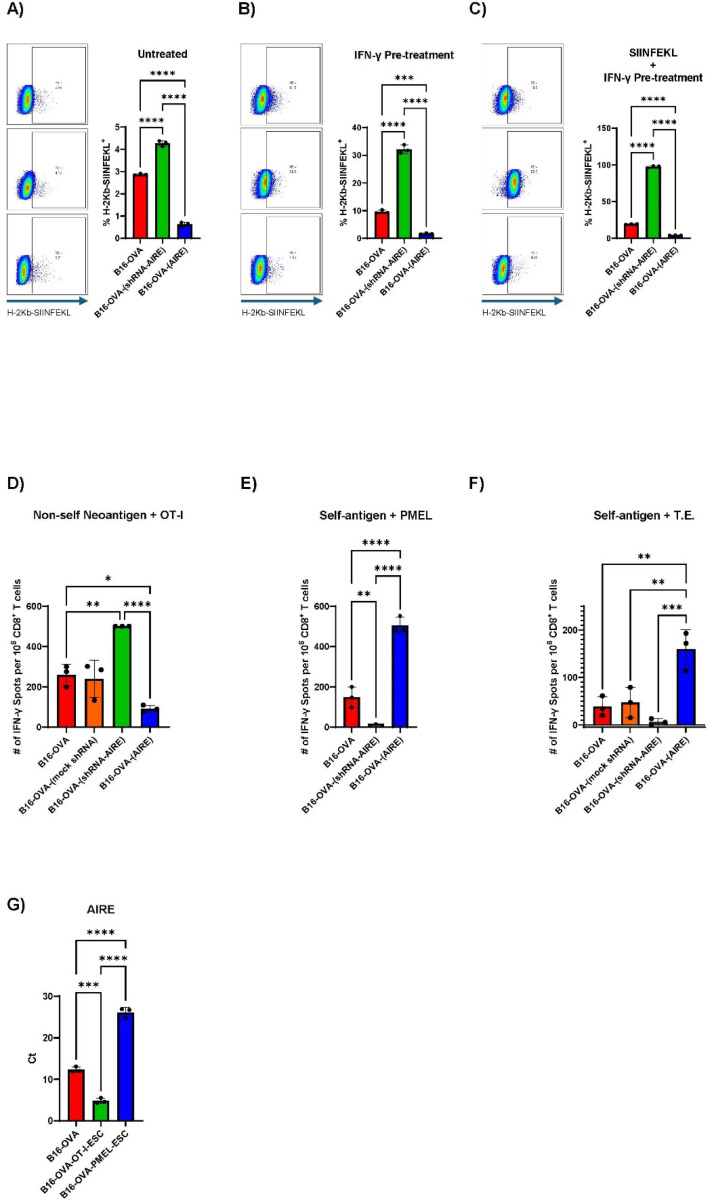
AIRE mediates MHC occupancy of SELF epitopes. **A.** Flow cytometry of B16-OVA, B16-OVA-(shRNA-AIRE) or B16-OVA-(AIRE) tumour cells labelled with the 25-D1.16 antibody which recognizes the H-2K^b^ MHC class I molecule bound to the OVA-derived peptide SIINFEKL (Kb-SIINFEKL). Histogram shows mean and standard deviation of three separate biological triplicates. **B/C**. The experiment of **A.** was repeated with the B16-OVA, B16-OVA-(shRNA-AIRE) or B16-OVA-(AIRE) tumour cells being pre-treated for 24 hours with 50 U of rIFN-γ (to increase surface expression of H-2K^b^ MHC class I molecules) (**B**) or with 50 U of rIFN-γ and 1 μg/mL of SIINFEKL peptide (**C**) prior to quantification of surface-expressed Kb-SIINFEKL by flow cytometry. Histograms show mean and standard deviation of three separate biological triplicates. **D.** 1×10^5^ B16-OVA B16-OVA-(scrambled shRNA), B16-OVA-(shRNA-AIRE) or B16-OVA-(AIRE) cells, pre-treated for 24 hours with 50 U of rIFN-γ, were co-cultured with 4 – 5 days *in vitro* activated OT-I CD8^+^ T cells at an Effector:Target ratio of 1:1 in 96-well ELISPOT plates (3 biological triplicates shown) for 48 hours at 37°C. OVA-specific IFN-γ positive spots were quantified by computer assisted image analyzer. **E/F.** The experiment of **D.** above was repeated with 4 – 5 days *in vitro* activated PMEL CD8^+^ T cells (**E**) or Tumour Experienced CD8^+^ T cells (T cells recovered from mice which had rejected B16-TK tumours in a CD8^+^ T cell dependent mechanism) (**F**) instead of OT-I T cells. **G.** 10^6^ B16-OVA cells, pre-treated for 24 hours with 50 U of rIFN-γ, were co-cultured with 4 – 5 days *in vitro* activated OT-I or PMEL CD8^+^ T cells at an Effector:Target ratio of 10:1 (3 biological triplicates shown) for 21 days at 37°C. Surviving B16-OVA cells were pooled and expanded for 2 weeks *in vitro* to give B16-OVA-OT-I-ESC or B16-OVA-PMEL-ESC populations. qrtPCR for levels of AIRE expression and GAPDH in B16-OVA, B16-OVA-OT-I-ESC or B16-OVA-PMEL-ESC cells are shown.

Knockdown of AIRE significantly increased levels of SIINFEKL presented by MHC Class I by B16-OVA-(shRNA-AIRE) cells compared to parental B16-OVA cells (Fig. 2A). In contrast, over-expression of AIRE reduced the levels of H2K^b^/SIINFEKL occupancy compared to parental B16-OVA cells (Fig. 2A). These effects were amplified when the B16-OVA cells were pre-treated with IFN-γ to increase levels of MHC Class I expression (Fig. 2B) and even further when additional SIINFEKL peptide was supplied along with IFN-γ pre-treatment (Fig. 2C). Thus, by changing levels of AIRE, the relative balance of SELF, AIRE-regulated, compared to non-AIRE-regulated, proteins available for MHC Class I occupancy can be re-set.

OT-I T cells are transgenic T cells with T Cell Receptor (TCR) specificity for the SIINFEKL epitope of OVA presented in the context of H-2K^b^ Class I MHC by B16-OVA tumour cells^53, 54^ (Fig. 2D). Whereas B16-OVA-(shRNA-AIRE) cells stimulated OT-I T cells significantly more than the parental B16-OVA cells, B16-OVA-(AIRE) cells were significantly less immunogenic to OT-I T cells. (Fig. 2D) confirming that MHC Class I occupancy by epitopes of the non-AIRE-mediated protein OVA was enhanced by lower levels of SELF epitopes (knockdown of AIRE) and inhibited by higher levels of SELF epitopes (AIRE over-expression). PMEL CD8^+^ T cells are transgenic T cells with TCR specificity for the H2-D^b^-restricted human gp100_25 – 33_ (hgp100, KVPRNQDWL) epitope but which will also be activated through their TCR by the murine homologue of the melanoma-associated antigen mgp100_25 − 33_ (mgp100, EGSRNQDWL)^37, 55^ (Fig. 2E). Gp100 is regulated by AIRE in the thymus^51^. Whereas B16-OVA-(shRNA-AIRE) cells were almost completely unable to stimulate PMEL T cells to secrete IFN-γ (Fig. 2E), unlike with OT-I T cells, B16-OVA-(AIRE) cells were significantly more immunogenic to PMEL T cells than were the parental B16-OVA cells (Fig. 2E). Thus, MHC occupancy by SELF, AIRE-regulated proteins — such as mgp100 — is enhanced by over-expression of AIRE and reduced by AIRE knockdown.

We have previously shown that clearance of B16-TK tumours (B16 tumour cells engineered to express the Herpes Simplex Virus thymidine kinase gene) by ganciclovir (GCV) is dependent upon CD8^+^ T cells, and that tumour-cured mice have CD8^+^ T cell responses (Tumour Experienced (T.E.) CD8^+^ T cells) against multiple B16 tumour associated antigens (TAA) ^56, 57, 58, 59^ and Fig. 2F. Similar to PMEL recognition of B16-OVA cell variants (Fig. 2E), B16-OVA-(shRNA-AIRE) cells were significantly less able to re-stimulate these T.E. CD8^+^ T cells to secrete IFN-γ (Fig. 2F) whereas B16-OVA-(AIRE) cells were significantly more immunogenic to T.E. CD8^+^ T cells than were the parental B16-OVA cells. (Fig. 2F), indicative that these T.E. anti-B16-TAA T cells had TCR specificities for SELF, AIRE-regulated proteins — such as mgp100, TYRP1, TYRP2.

We also observed that B16-OVA cells which escaped OT-I T cells *in vitro* expressed significantly higher levels of AIRE than the parental B16-OVA prior to T cell pressure (Fig. 2G). In contrast, B16-OVA cells which escaped from PMEL T cell pressure expressed significantly decreased levels of AIRE compared to parental B16-OVA (Fig. 2G) — indicating that changes in AIRE expression can be used to escape from T cell pressure in a manner heavily dependent upon the nature of the antigen targeted by T cells (AIRE-regulated or not).

### AIRE mediated regulation of TAA can be exploited for adoptive T cell therapies.

**Figure 3:**
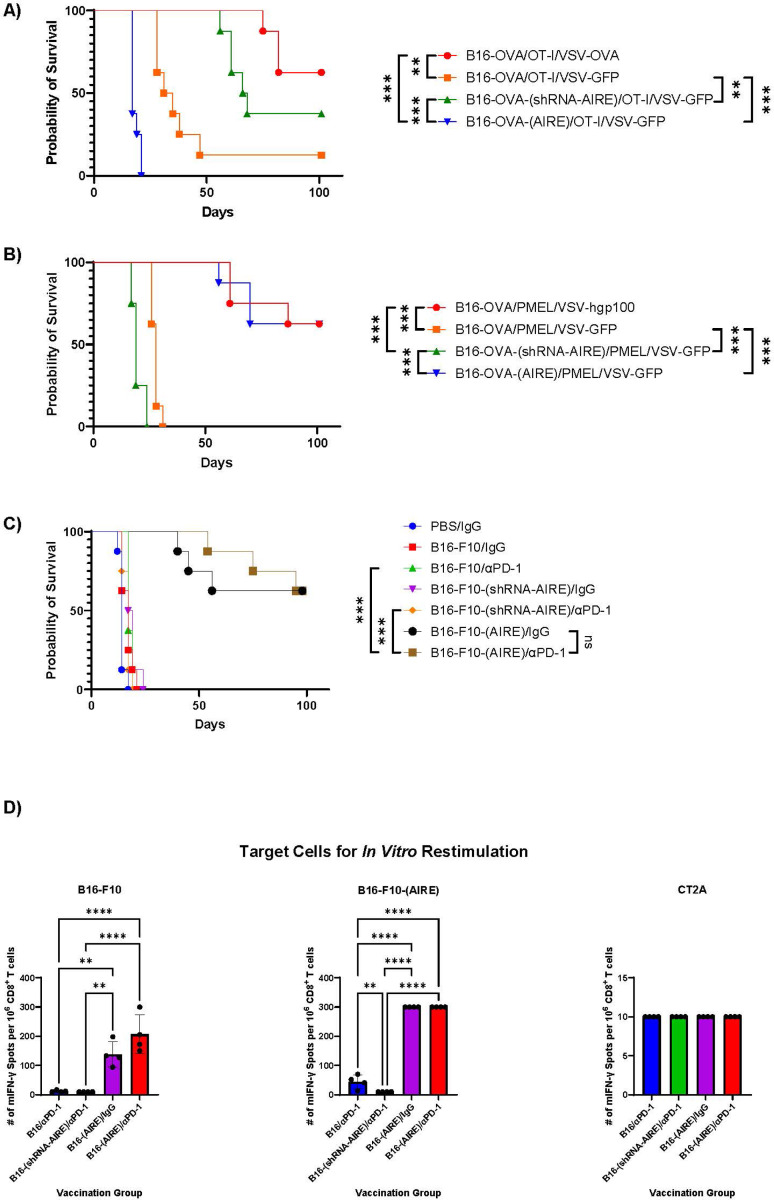
AIRE-mediated alterations in the epitope profile of tumour cells can be exploited for T cell therapy of tumours. **A/B.** C57Bl/6 mice with 10 days established subcutaneous B16-OVA, B16-OVA-(shRNA-AIRE) or B16-OVA-(AIRE) tumours were treated intravenously with 10^7^ naïve OT-I (**A**) or PMEL (**B**) CD8^+^ T cells. 2 days later (d12), mice were treated with VSV-OVA, VSV-hgp100 or VSV-GFP as shown (5×10^6^ pfu virus/injection, i.v.). This cycle of adoptive T cell transfer and virus boost was repeated on days 14 and 17 and 19 and 21. Tumour diameters were measured thrice weekly in two dimensions using calipers, and mice were sacrificed when tumour size was ∼1.0 × 1.0 cm in two perpendicular directions. Survival with time is shown. **C.** C57Bl/6 mice with 10 days-established B16-F10, B16-F10-(shRNA-AIRE) or B16-F10-(AIRE) tumours were treated with dendritic cells loaded *in vitro* with lysates of PBS (no lysate) B16-F10-(shRNA-AIRE) or B16-F10-(AIRE) cells (10^6^ DC loaded with lysate equivalent of 10^7^ tumour cells per injection) (see Materials and Methods) on days 10,12 & 14. Mice were then treated with anti-PD-1 antibody or isotype IgG control as shown on days 17, 19, 21 and 24, 26 & 28. Survival (tumour size) with time is shown. **D.** CD8^+^ T cells (10^6^/well) were isolated from mice at endpoint in **C.** (4/group) (see Materials and Methods) which had been treated with DC vaccines loaded with lysates of B16-F10, B16-F10-(shRNA-AIRE) or B16-F10-(AIRE) and co-treated with anti-PD-1 or control IgG, as shown. CD8^+^ T cells were re-stimulated in 96-well ELISPOT plates (4 biological triplicates shown) with 5×10^5^ live target cells (B16-F10, B16-F10-(AIRE) or CT2A (pre-treated with IFN-γ for 24 hours to increase MHC class I expression) for 48 hours at 37°C. Tumour-specific IFN-γ positive spots were quantified by computer assisted image analyzer.

B16-OVA tumours are highly susceptible to adoptive transfer of naïve OT-I or PMEL T cells when the OT-I/PMEL T cells are activated *in vivo* by co-infection with the immunogenic Vesicular Stomatitis Virus (VSV) expressing either OVA or hgp100^60, 61^ (Figs. 3A&B). However, when the naïve transgenic T cells were not supported by the VSV-TAA adjuvant (replaced by VSV-GFP), therapy of established B16-OVA tumours was significantly reduced^60, 61^ (Figs. 3A&B). However, tumours formed by B16-OVA-(shRNA-AIRE) cells were treated very effectively by transfer of naïve OT-I T cells in the absence of *in vivo* activation by VSV-OVA (Fig. 3A), suggesting that reduced levels of AIRE were associated with both increased levels of (non-AIRE regulated) OVA/SIINFEKL target antigen and the ability of these cells to activate naïve T cells *in vivo*. Consistent with the *in vitro* data of Fig. 2, the converse was true for treatment with PMEL T cells where tumours derived from B16-OVA-(AIRE) cells were recognized and cleared as effectively by naïve PMEL T cells as if they had been activated *in vivo* by VSV-hgp100 (Fig. 3B). Taken together Figs. 3A&B show that *in vivo* modulation of AIRE expression in tumours can be used to enhance adoptive T cell therapies with a close dependency upon whether the T cell target antigen is AIRE regulated or not.

### AIRE-mediated regulation of TAA can be exploited for vaccine-based immunotherapy

We asked whether changes in AIRE expression in tumour cells, either through local delivery or in the vaccine setting, would generate T cell responses which can target parental tumours not modified for AIRE -over- or -under-expression. First, we tested the underlying hypothesis that changes in the constellation of epitopes presented by AIRE-modified tumour cells can be faithfully transferred through a dendritic cell vaccine to the activation of *de novo* T cell responses against the modified epitope profile which would also include heteroclitic T cell specificities against a subset of TAA expressed by the parental, non-AIRE-modified tumours. B16-F10 tumours were almost completely unresponsive to immune checkpoint blockade (ICB) with anti-PD-1 even with dendritic cell-B16-F10-, or B16-F10-(shRNA-AIRE)-loaded vaccines (Fig. 3C). In contrast, dendritic cells loaded with lysates of B16-F10-(AIRE) cells were extremely effective at vaccinating against established B16-F10 tumours either alone, or in combination with, anti-PD-1 ICB (Fig. 3C). Very low levels of B16-F10-reactive CD8^+^ T cells were detected from spleens of mice treated with anti-PD-1 ICB and a dendritic cell vaccine loaded with lysates of B16-F10 cells when they were re-stimulated with B16-F10-(AIRE) target cells (Fig. 3D), suggesting that over-expression of AIRE exposes higher levels of epitopes which can be targeted by low levels of CD8^+^ T cells stimulated *in vivo* by vaccination with B16-F10 + ICB. In contrast, significantly higher levels of B16-F10-reactive CD8^+^ T cells were present in spleens of mice treated with dendritic cells loaded with lysates of B16-F10-(AIRE) cells both without, and with, combination ICB therapy (Fig. 3D). Higher numbers of reactive CD8^+^ T cells were observed in spleens from those groups when re-stimulated with B16-F10-(AIRE) target cells (Fig. 3D). There was no significant cross reactivity of these B16-F10-reactive T cells induced by over-expression of AIRE in B16-F10 cells with an unrelated CT2A glioma cell line of the same MHC background (Fig. 3D).

Therefore, the constellation of epitopes presented by DC loaded with B16-F10-(AIRE) lysates raised heteroclitic T cell responses which could cross-react against parental B16-F10 tumours which were present amongst a larger population of B16-F10-(AIRE)-specific CD8^+^ T cells.

### AIRE in Diffuse Midline Gliomas

**Figure 4:**
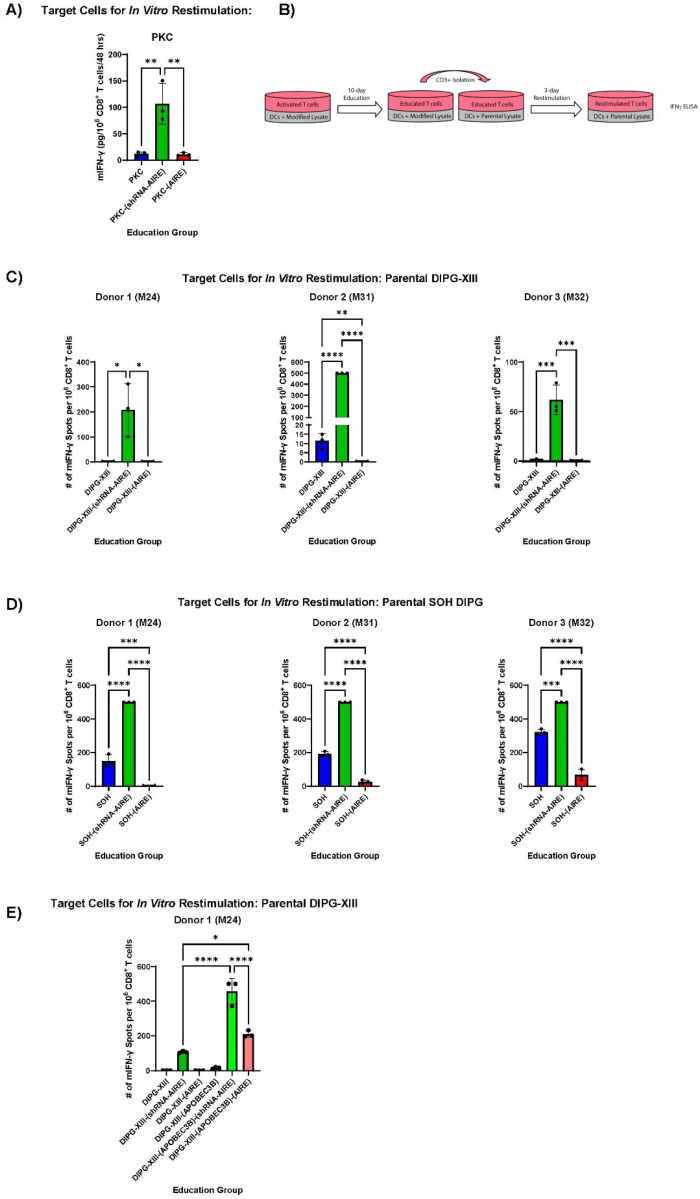
AIRE maintains SELFNESS of Diffuse Midline Gliomas. **A.** Splenocytes from naïve C57Bl/6 mice were co-cultured with live PKC, PKC-(shRNA-AIRE) or PKC-(AIRE) cells, pre-treated for 24 hours with IFN-γ to enhance MHC Class I presentation, at a ratio of 10:1 for three days with IL-2. On days 6/7 and 9/10, co-cultures were re-plenished with live, IFN-γ-pre-treated PKC variant tumour cells. After 2 weeks of *in vitro* priming with PKC, PKC-(shRNA-AIRE) or PKC-(AIRE) cells, purified CD8^+^ T cells were co-cultured with 10^5^ parental PKC tumour cells at a ratio of ~10:1. Cell-free supernatants were collected after 48 hours and tested by ELISA for IFN-γ. Mean of three biological triplicates are shown. **B.** *In vitro* protocol for priming/educating human CD8^+^ T cells with human Diffuse Midline Glioma cell variants (see Materials and Methods for details). **C/D.** Human CD8^+^ T cells from three different donors were primed with human DMG DIPG-XIII (**C**) or SOH (**D**) cell lines as shown in **B.** CD8^+^ T cells were re-stimulated in 96-well ELISPOT plates (3 biological triplicates shown) with live, parental DIPG-XIII (**C**) or SOH (**D**) target cells respectively (pre-treated with IFN-γ for 24 hours to increase MHC class I expression) for 72 hours and tumour-specific IFN-γ positive spots quantified as shown. **E.** The experiment of **C.** was repeated with priming of donor CD8^+^ T cells by DIPG-XIII, DIPG-XIII-(AIRE), DIPG-XIII-(shRNA-AIRE), DIPG-XIII-(APOBEC3B), DIPG-XIII-(APOBEC3B)-(AIRE) or DIPG-XIII-(APOBEC3B)-(shRNA-AIRE) cells and re-stimulation in IFN-γ ELISPOT plates for 3 days with parental DIPG-XIII cells.

Murine PKC cells, derived from a K27M mutant genetically engineered model of Diffuse Midline Glioma, are very poorly immunogenic in C57Bl/6 mice and could not induce IFN-γ secretion from CD8^+^ T cells even after extensive *in vitro* priming/education (Fig. 4A). PKC-(CMV-AIRE) cells were equally non-immunogenic (Fig. 4A). In contrast, PKC-(shRNA-AIRE) cells stimulated low, but significant, levels of IFN-γ from CD8^+^ T cells against parental PKC cells following *in vitro* priming (Fig. 4A). Similarly, knockdown of AIRE in two different human DMG cell lines significantly enhanced priming of human CD8^+^ T cells against the parental cells (Fig. 4B,C&D). Of 5 donors tested, two generated very potent allogeneic T cell responses (IFN-γ > 500 IFN-γ spots per well) against the DIPG-XIII cell line upon co-culture of DIPG-XIII cells with donor CD8^+^ T cells (no priming/education phase (Fig. 4B)). Of three different donors where minimal allogeneic reactivity was observed, over-expression of AIRE could not enhance immunogenicity (Fig. 4C). However, when AIRE expression was knocked down, priming/education of CD8^+^ T cells against the unmodified parental DIPG-XIII cell line was now possible (Fig. 4C). Priming/education with parental SOH DMG cells generated significant CD8^+^ T cell reactivity against the parental SOH cells (Fig. 4D). Over-expression of AIRE significantly inhibited the ability of the SOH tumour cells to prime CD8^+^ T cells against themselves (Fig. 4D). Conversely, priming with SOH cells in which AIRE was knocked down uncovered a more potent T cell response against the parental tumour cells (Fig. 4D).

Transduction of tumour cells with the cytidine deaminase APOBEC3B induces genome mutations which generate immunogenic neo-epitopes in both human and murine tumour model systems^42^. To test the effects of AIRE expression on priming against DIPG cells expressing a higher mutational load than in the parental cells, DIPG-XIII cells transduced with an APOBEC3B-expressing vector^42^ were tested in the assay of Fig. 4B&C. As before, only DIPG-XIII-(shRNA-AIRE) cells effectively educated CD8^+^ T cells to recognize the parental DIPG-XIII tumour cells (Fig. 4E). APOBEC-modified/mutated DIPG-XIII-(APOBEC3B) cells were very slightly, but significantly, more immunogenic than the un-mutated DIPG-XIII cells, presumably due to an increased mutational load generating immunogenic epitopes^42^ (Fig. 4E). Increased AIRE expression in APOBEC-modified/mutated cells was significantly more effective in priming T cell responses against parental DIPG-XIII cells than the DIPG-XIII-(AIRE) cells (Fig. 4E) — suggesting that an increased mutational load can generate potentially heteroclitic neo-epitopes in AIRE-regulated proteins. Moreover, knock down of AIRE in DIPG-XIII-(APOBEC3B) cells generated the most potent CD8^+^ T cell responses against parental DIPG-XIII cells of all the DIPG-XIII cell variants (Fig. 4E) — suggesting also that (APOBEC3B-induced) neo-epitopes in non-AIRE-regulated genes are produced and are potentially highly heteroclitic and immunogenic. Taken together, these data suggest that AIRE routinely maintains the high levels of SELFNESS of DMG tumours and that by inhibiting AIRE, and/or introducing a higher mutational load, these tumours may become more amenable to T cell mediated immunotherapy.

### Inhibition of AIRE expression enables immunotherapy of Diffuse Midline Glioma.

**Figure 5:**
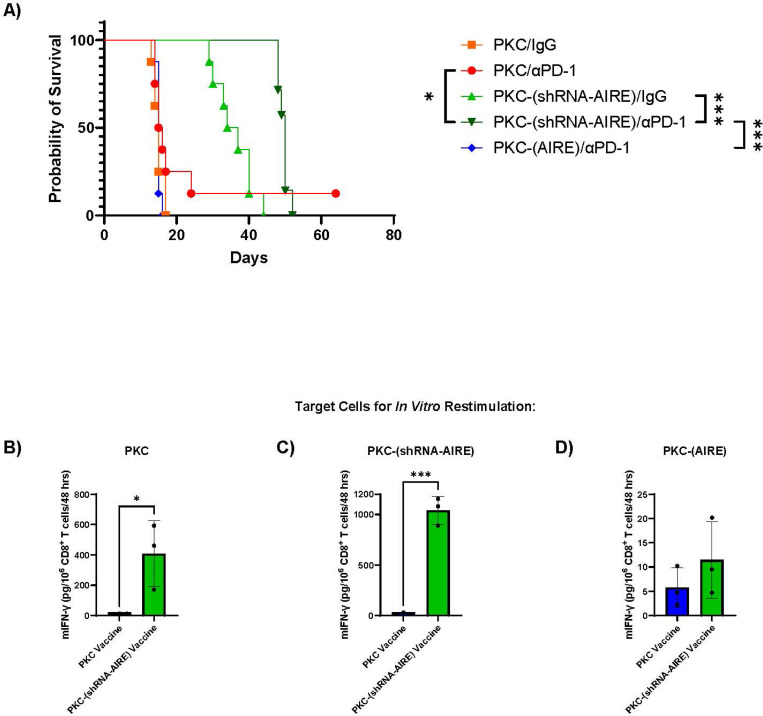
Inhibition of AIRE expression reveals *de novo* T cell responses against Diffuse Midline Glioma. A. C57Bl/6 mice with 5 days-established PKC tumours were treated with dendritic cells loaded *in vitro* with lysates of PKC cells alone, PKC-(shRNA AIRE) or PKC-(AIRE) cells (10^6^ DC loaded with lysate equivalent of 10^7^ tumour cells per injection) (see Materials and Methods) on days 5, 7 & 9. Mice were then treated with anti-PD-1 antibody or isotype IgG control as shown on days 12, 14, 16 and 19, 21 & 23. Survival (tumour size) with time is shown. **B-D.** CD8^+^ T cells (10^6^/well) were isolated from mice at endpoint in **A.** (3/group) (see Materials and Methods) which had been treated with DC vaccines loaded with lysates of parental PKC cells or with DC vaccines loaded with lysates of PKC-(shRNA-AIRE) cells and were re-stimulated with 5×10^5^ live target cells PKC (**B**); PKC-(shRNA-AIRE) (**C**); or PKC-(AIRE) (**D**) cells (pre-treated with IFN-γ for 24 hours to increase MHC class I expression) for 48 hours at 37°C (3 biological triplicates per group) and IFN-γ in the supernatants was measured.

Typical of DMG, PKC tumours were both non immunogenic themselves in the context of dendritic cell vaccination and were almost completely unresponsive to immune checkpoint blockade (ICB) with anti-PD-1 (Fig. 5A). Dendritic cells loaded with lysates from PKC-(AIRE) cells were also unable to immunize against PKC tumours, confirming that AIRE over-expression was not revealing new SELF epitopes for *de novo* T cell recognition even with ICB (Fig. 5A). However, dendritic cells loaded with lysates of PKC-(shRNA-AIRE) cells significantly improved survival times of PKC tumour-bearing mice, an effect which was significantly further improved in combination with anti-PD-1 ICB (Fig. 5A). CD8^+^ T cells from spleens of mice treated with dendritic cell vaccines loaded with lysates of PKC parental cells did not recognize parental PKC, PKC-(shRNA-AIRE) or PKC-(AIRE) cells as targets upon re-stimulation *in vitro* (Figs. 5B–D). However, consistent with Fig. 5A, CD8^+^ T cells from spleens of mice treated with dendritic cell vaccines loaded with lysates of PKC-(shRNA-AIRE) cells could be re-stimulated with both PKC-(shRNA-AIRE) cells themselves and by parental PKC cells (although to a lesser degree) (Figs. 5B&C). Although vaccination with PKC-(shRNA-AIRE) cells raised *de novo* T cell responses against parental PKC cells (Fig. 5B), those T cells were unable to be re-stimulated *in vitro* with PKC-(AIRE) cells (Fig. 5D) — implying that levels (as well as identity) of SELF proteins/epitopes expressed by DMG may be important in determining SELFNESS (lack of T cell immunogenicity). Overall, Figs. 4&5 suggest that expression of AIRE plays a key role in the inhibition of T cell recognition of DMG cells and that by inhibiting AIRE novel T cell targets for immune attack on these tumours may be revealed.

### In vivo delivery of AIRE stimulates potent T cell-mediated tumour rejection.

**Figure 6:**
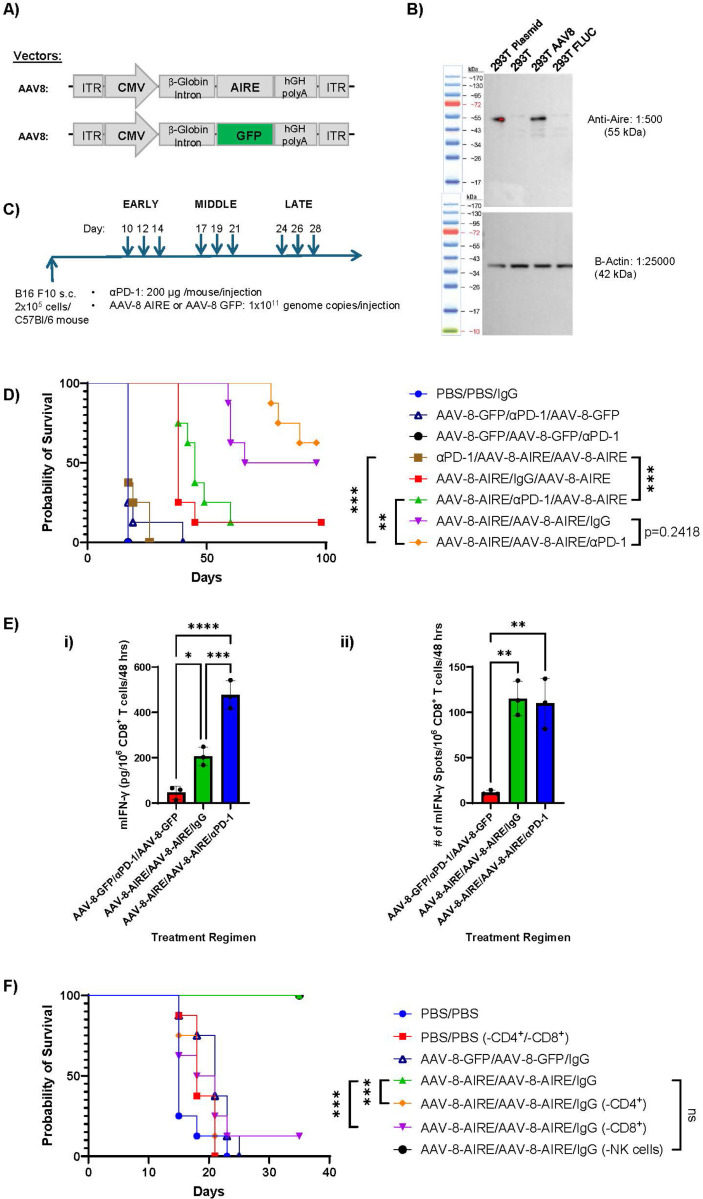
*In vivo* delivered AIRE cures established tumours. **A.** Schematic of AAV-8-AIRE and AAV-8-GFP vectors. **B.** Tumour cells were transfected with CMV-AIRE plasmid (lane 1), left un-transfected (lane 2) or infected with AAV-8-AIRE (lane 3) or AAV-8-Control (lane 4) (1×10^9^ genome copies). 48 hours later, cells were harvested and assayed by Western Blot for AIRE and β-Actin. **C/D.** 5 days established B16-F10 subcutaneous tumours were injected in three cycles EARLY 5, 7, 9; MIDDLE 12, 14, 16; and LATE 19, 21, 23 with either virus (AAV-8-AIRE or AAV-8-GFP) or with anti-PD-1 ICB antibody or control IgG antibody (200 μg/injection) (**C**). When tumours reached an endpoint of 1.0 cm tumour diameter in any direction, mice were euthanized and survival by tumour size endpoint is shown (**D**). **E.** Spleens were recovered from the first 3 mice in each group to be euthanized. CD8^+^ T cells were isolated by magnetic bead sorting and co-cultured at an Effector:Target ratio of 5:1 in 96-well ELISPOT plates with live target B16-F10 cells, pre-treated for 24 hours with IFN-γ to increase MHC Class I expression for 48 hours at 37°C. Levels of IFN-γ were measured from the supernatants by ELISA (i) and B16-F10-specific IFN-γ ELISPOT positive spots (ii) (three biological replicates per group) were quantified by computer assisted image analyzer. **F.** 5 days established B16-F10 subcutaneous tumours were injected intra-tumorally on days 5, 7, 12, 14, 16 with PBS or virus (AAV-8-AIRE or AAV-8-GFP) and with control IgG antibody or depleting antibody (anti-CD4, anti-CD8 or anti-NK) (200 μg/injection ip) on days 5, 7, 12 &14. When tumours reached an endpoint of 1.0 cm tumour diameter in any direction, mice were euthanized and survival by tumour size endpoint is shown.

An AAV-8-AIRE vector led to high levels of AIRE transduction of cells *in vitro* (Fig. 6A&B) and we used this vector to test direct *in vivo* delivery of AIRE in combination with anti-PD-1 ICB could generate therapy by inducing *de novo* T cell responses which either early or late ICB would be able to augment (Fig. 6C). Anti-PD-1 ICB prior to AIRE delivery was completely ineffective (Fig. 6D). *In vivo* delivery of early AAV-8-AIRE was significantly more therapeutic against established B16-F10 tumours than the PBS control, AAV-GFP, or anti-PD-1 ICB treatment (Fig. 6D). Addition of anti-PD-1 ICB subsequent to early AAV-8-AIRE gave a trend towards improved survival which was not significant (Fig. 6D). However, multiple injections of AAV-8-AIRE alone, led to significant numbers of tumour cures (Fig. 6D). Addition of subsequent anti-PD-1 ICB significantly improved time of survival but did not reach significance in overall cure rate (Fig. 6D). All 9 mice which were tumour free at day 96 following treatment with AAV-8-AIRE in Fig. 6D, with or without anti-PD-1 ICB, rejected a subsequent challenge with 2×10^5^ parental B16-F10 cells, whereas 5 control mice succumbed to tumour by Day 25 — showing generation of immunological memory by AAV-8-AIRE therapy. Consistent with this, ELISPOT analysis showed that very similar numbers of anti-B16-F10 CD8^+^ T cells were generated by AAV-8-AIRE therapy alone as by AAV-8-AIRE + anti-PD-1 ICB (Fig. 6E). However, the activity of those CD8^+^ T cells (amount of IFN-γ/CD8^+^ T cell) was significantly greater in mice which had received anti-PD-1 ICB in addition to the AAV-8-AIRE treatment (Fig. 6E). The relevance of these anti-B16-F10 CD8^+^ T cell responses to the overall therapy in Fig. 6D was confirmed by antibody depletion studies which showed that AAV-8-AIRE-mediated therapy of B16-F10 tumours was dependent upon both CD4^+^ and CD8^+^ T cells, but not NK cells, (Fig. 6F).

### AIRE over-expression in melanoma cells induces a novel set of epitope expression.

**Figure 7:**
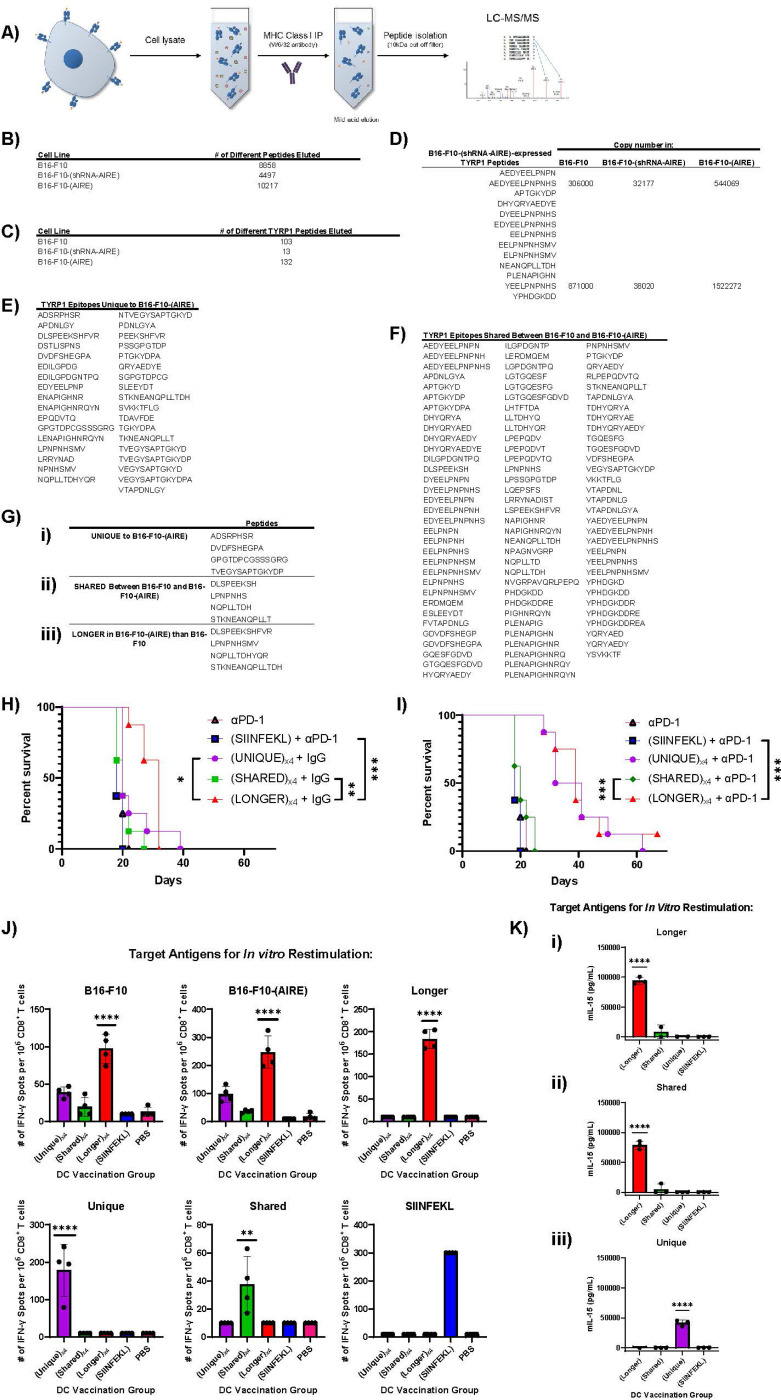
AIRE overexpression generates both a greater number and diversity of epitopes. **A.** Schematic for MHC Class I immunoprecipitation from B16-F10 cells using the anti-H-2K^b^ antibody (Clone Y-3). **B.** The total number of different peptides eluted from B16-F10 parental, B16-F10-(AIRE) or B16-F10-(shRNA-AIRE) cells is shown. **C.** The total number of different TYRP1 peptides eluted from B16-F10 parental, B16-F10-(AIRE) or B16-F10-(shRNA-AIRE) cells is shown. **D.** The 13 TYRP1 peptides eluted from B16-F10-(shRNA-AIRE) cells are shown. All these peptides were also eluted from both B16-F10 parental and B16-F10-(AIRE) cells but in higher abundances as shown for two representative peptides. **E/F.** TYRP1 epitopes uniquely eluted from B16-F10-(AIRE) cells (**E**) and those shared between B16-F10 and B16-F10-(AIRE) cells (**F**) are shown **G.** Four peptides which were i) uniquely eluted from B16-F10-(AIRE) cells, ii) shared between B16-F10 and B16-F10-(AIRE) cells or iii) exclusively eluted from B16-F10-(AIRE) cells as longer versions of those in ii) were synthesized and mixed at 0.25 μg per peptide for loading onto DC for the experiments of **H/I. H/I.** C57Bl/6 mice with 5 days-established B16-F10 tumours were treated with dendritic cells loaded *in vitro* with SIINFEKL peptide (1 μg per 10^6^ DC/injection) with PBS or with peptide sets of the Unique, Shared, Longer peptides (see **G.** above) (at 0.25 μg per peptide, total 1 μg per 10^6^ DC/injection) on days 5, 7 & 9. Mice were then treated with anti-PD-1 antibody or isotype IgG control as shown on days 12, 14, 16 and 19, 21, 23. For clarity, survival (tumour size) with time is shown for groups treated with control IgG (no ICB) (**H**) or with ICB (**I**) (**H**&**I** are the same experiment). **J.** CD8^+^ T cells (10^6^/well) were isolated from mice at endpoint in **H/I**. (4/group) which had been treated with DC vaccines loaded with different peptides and co-treated with anti-PD-1 as shown. 10^6^ CD8^+^ T cells were re-stimulated in 96-well ELISPOT plates (4 biological triplicates shown) with either 5×10^5^ live target cells (B16-F10 or B16-F10-(AIRE) (pre-treated with IFN-γ for 24 hours to increase MHC class I expression) or with either 5×10^5^ dendritic cells loaded with the Unique, Shared, Longer peptide sets or SIINFEKL peptide (total of 1 μg peptide/10^6^ DC) for 48 hours at 37°C. IFN-γ positive spots were quantified by computer assisted image analyzer. **K.** CD4^+^ T cells were isolated by magnetic bead sorting from mice at endpoint in **H/I** (3/group) which had been treated with DC vaccines loaded with different peptides and co-treated with anti-PD-1 as shown. 5×10^5^ CD4^+^ T cells were re-stimulated in 96-well plates (3 biological triplicates shown) with 5×10^5^ dendritic cells loaded with the Longer (i), Shared (ii), or Unique (iii) peptide sets (total of 1 μg peptide/10^6^ DC) for 48 hours at 37°C. 48 hours later IL-15 in the supernatant was measured by ELISA (biotechne, R&D Systems, Mouse IL-15 DuoSet ELISA, Catalog #: DY447).

Mass spectrometry of the peptides eluted from the MHC class I molecules of B16-F10, B16-F10-(shRNA-AIRE) and B16-F10-(AIRE) cells (Fig. 7A) identified a total of 8858, 4497 and 10217 peptides respectively (Fig. 7B), suggesting that AIRE is a major controller of the absolute number of MHC-Class I-presented epitopes. Changes in the total numbers of epitopes from the well characterized melanoma TAA TYRP1 between B16-F10 cell lines expressing different levels of AIRE (Fig. 7C) reflected the overall data set (Fig. 7B) in that over-expression of AIRE in B16-F10 cells increased the total number of TYRP1 epitopes, whilst knock down of AIRE dramatically decreased the number of TYRP1 epitopes (Fig. 7C). Within this data set, we observed three classes of TYRP1 epitopes which varied between the B16-F10, B16-F10-(shRNA-AIRE) and B16-F10-(AIRE) cell lines. In the first, some TYRP1 epitopes were shared between all three lines, but were more abundantly present in the B16-(AIRE) cells (Fig. 7D). All 13 TYRP1 peptides expressed in the B16-F10-(shRNA-AIRE) line were shared between all three cell lines (Fig. 7D) but were present at the lowest abundances. The second class of TYRP1 epitopes were those that were unique to one cell line. In this respect, 35 peptides were unique to B16-F10-(AIRE) cells and not expressed by either parental B16-F10 or B16-F10-(shRNA-AIRE) cells (Fig. 7E), whereas 97 peptides were eluted from both B16-F10-(AIRE) and B16-F10 parental cells (Fig. 7F). Only 6 peptides were exclusively present in B16-F10 parental cells. The third class of TYRP1 peptides comprised those peptides that shared a core sequence between B16-F10 parental and B16-F10-(AIRE) cells but which were 1–3 amino acids longer in the peptides eluted from the B16-F10-(AIRE) line usually at the Carboxy terminal ends of the respective peptides (examples in Fig. 7G).

To investigate the biological relevance of these three different classes of TYRP1 peptides, we tested 4 of each of the Shared, B16-F10-(AIRE)-Unique and B16-F10-(AIRE)-Longer class (Fig. 7G) as vaccines against parental B16-F10 tumours. Dendritic cell vaccines loaded with either the Shared, or Unique peptides were not significantly more effective at slowing the growth of B16-F10 tumours than controls (DC loaded with PBS or SIINFEKL peptide) (Fig. 7H). However, treatment with dendritic cells loaded with the Longer peptide set significantly increased median survival times of B16-F10 tumour bearing mice compared to controls (Fig. 7H). In combination with anti-PD-1 ICB, both the Longer and the Unique peptide set, but not the Shared peptide set, significantly enhanced survival times of B16-F10 tumour bearing mice compared to control treatments (Fig. 7I). Only treatment with either the Unique or the Longer peptide sets primed recall responses against B16-F10 parental cells which were significantly greater than the control treatments, with vaccination with the Longer peptides being significantly greater than any other treatment (Fig. 7J). The same pattern was observed with the recall response of CD8^+^ T cells against B16-(AIRE) targets *in vitro* but at higher magnitudes of IFN-γ secretion (Fig. 7J) confirming that the Longer and Unique peptide sets contain AIRE-induced T cell targets which generate T cell responses which can cross react back against parental B16-F10 cells.

A key observation form Fig. 6F was that AIRE-mediated tumour therapy was dependent upon CD4^+^, as well as CD8^+^, T cells. Consistent with a critical role for IL-15-mediated dendritic cell activation by CD4^+^ T cell help^62, 63, 64, 65^ in this therapy, CD4^+^ T cells from spleens of mice treated with DC/Longer + aPD-1 in Fig. 7I not only induced strong IL-15 responses *in vitro* from DC loaded with the Longer peptides (Fig. 7Ki) but also from DC loaded with the shorter epitopes which were shared between B16-F10-(AIRE) and B16-F10 parental cells (Fig. 7Kii). CD4^+^ T cells following vaccination with DC/Shared + aPD-1, which was therapeutically ineffective (Fig. 7I), could not activate DC to produce IL-15 even when the DC presented the Shared peptides as targets except from a single mouse (Fig. 7Kii). CD4^+^ T cells from mice vaccinated with DC/Unique + aPD-1 were able to activate weak IL-15 responses from DC loaded with the Unique peptides suggesting that these B16-(AIRE) expressed Unique epitopes generated in AIRE-over-expressing cells may contain T helper functions as well (Fig. 7Kiii). As expected, CD4^+^ T cells recovered from mice treated with DC/SIINFEKL + aPD-1 in Figs. 7H/I did not induce IL-15 secretion when co-cultured with DC loaded with any of the peptide sets. These data show that AIRE-mediated changes in epitope display by B16-F10 tumour cells can lead to the provision of CD4^+^ T cell helper epitopes capable of activating dendritic cells against both AIRE-specific and parental expressed epitopes of TAA such as TYRP1.

## DISCUSSION

AIRE induces expression of a wide range of peripheral tissue specific antigens (TSA) principally in medullary thymic epithelial cells (mTEC)^1, 3, 4, 5, 7^. We show here that changes in the levels of AIRE expression — either increased or decreased — in both high mutational load, immunogenic melanomas as well as in low mutational load, poorly immunogenic DMG cells led to profound alterations in their immunogenicity, visibility to T cells and enhanced tumour rejection by T cell mediated therapy. In contrast to mTEC in the thymus which display an array of SELF epitopes to encourage T cell activation and negative selection^3, 5, 12^, we hypothesized that AIRE expression in tumour cells sets a baseline level of SELFNESS such that there is minimal anti-tumour T cell reactivity. By dialing the levels of AIRE up or down, that steady state SELFNESS is altered, revealing a new profile of epitopes — both qualitatively and quantitatively — presented by the tumour cells, allowing for *de novo* T cell reactivities to be generated *in vivo*. Our data show that amongst those *de novo* T cell reactivities there exists at least a subset of both helper CD4^+^ and heteroclitic CD8^+^ effector T cells^36, 37, 55^ which can cross react between AIRE-induced epitopes and similar epitopes on the parental tumour cells, leading to potent tumour rejection responses.

Both murine and human tumour cells express AIRE in the two different tumour types, melanoma and Diffuse Midline Glioma (DMG), which have been the focus of our clinical trials^66, 67^ (Fig. 1A). In the B16-OVA cell line, the transfected, CMV-controlled OVA was independent of AIRE expression (Fig. 1). However, despite not being AIRE-regulated, levels of presentation of the SIINFEKL epitope of OVA by B16-OVA tumour cells were increased by lowering AIRE expression (Figs. 2A–C). One interpretation of these data is that by decreasing AIRE levels fewer epitopes from SELF, AIRE-regulated proteins are available for MHC Class I occupancy, leading to higher levels of Class I MHC-bound epitopes derived from non-AIRE mediated proteins — such as SIINFEKL/OVA. Similarly, by increasing AIRE levels in B16-OVA cells fewer epitopes of the non-AIRE regulated OVA protein become available for presentation by MHC Class I molecules and are replaced by epitopes from SELF, AIRE-regulated proteins.

Manipulation of AIRE levels in B16-OVA cells (by either knock down or over-expression) effectively enhanced their immunogenicity (T cell visibility) for TCR-mediated T cell therapies (Figs. 2D–G & 3) dependent upon the nature of the TAA being targeted (AIRE regulated or not). Inhibition of AIRE in B16-OVA tumours promoted greater visibility to OT-I T cells by increasing presentation of OVA/SIINFEKL epitopes (non-AIRE regulated) relative to AIRE controlled SELF epitopes. Conversely, increased AIRE expression in B16-OVA cells correlated with increased T cell therapy with PMEL T cells, presumably by enhancing levels of the AIRE-regulated, PMEL-recognized SELF epitope of gp100 (Figs. 2&3). Therefore, manipulation of AIRE levels in tumour cells can be used to enhance their immunogenicity for TCR-mediated T cell therapies, although the nature of the tumour antigen being targeted (AIRE regulated or not) is a critical factor in whether AIRE levels should be enhanced or decreased to achieve better T cell killing. In addition, our data show that tumour cell intrinsic modulation of AIRE expression is one mechanism by which tumour cells can evolve to escape T cell killing (Fig. 2G) and that the nature of the antigen targeted by the T cells is a key factor in whether selection of tumour cells expressing higher, or lower, AIRE levels is induced to achieve that escape. Therefore, AIRE expression in tumours may be a biomarker of escape from immunotherapy by altering the profile of epitopes that are being targeted by the therapy ^15, 16^.

We investigated whether, in the absence of available T cells targeting a specific known TAA, changes in AIRE expression in tumour cells could generate heteroclitic T cell responses^36, 37, 55^ which could target tumours not modified with AIRE-over-, or -under, expression. Our underlying hypothesis was that changes in the constellation of epitopes presented by AIRE-modified tumour cells could be faithfully transferred through antigen presentation (either by antigen presenting cells (APC) or the AIRE-modified tumour itself) to the activation of *de novo* T cell responses against the modified epitope profile; in turn, these *de novo* T cell responses would include heteroclitic T cell specificities^36, 37, 55^ which could also recognize a subset of TAA expressed by the parental, non-AIRE-modified tumours. Figures 3C&D show that the profile of epitopes presented by DC loaded with B16-F10-(AIRE) lysates raised T cell responses which could very effectively cross-react back onto the profile of epitopes expressed by the parental B16-F10 tumours, leading to significant numbers of tumour cures (Fig. 3C). Those B16-F10-specific heteroclitic responses existed amongst a larger population of CD8^+^ T cells which also recognized B16-F10-(AIRE)-specific targets (Fig. 3D).

Figures 4A–E show that AIRE routinely maintains the high levels of SELFNESS (lack of immunogenicity) of DMG in both murine and human tumours. In contrast to B16 melanomas, knocking down AIRE expression in DMG uncovered novel T cell targets for immune attack. DMG tumours, characterized by the K27M mutation inducing global hypomethylation and a state of open chromatin throughout the genome, have very low mutational loads, and are very poorly infiltrated with immune cells, possibly associated with their development at early ages and in children with very naïve T cell repertoires. Increasing the mutational load carried by the DMG cells using APOBEC3B mutation^42^ further reduced SELFNESS to a very moderate degree (Fig. 4E). With this APOBEC3B-induced increased mutational load, increasing levels of AIRE enhanced T cell reactivity to DMG cells — probably through the generation of novel neo-epitopes in AIRE regulated genes, which generated T cell responses against un-mutated epitopes on the parental DMG cells (Fig. 4E). Finally, by increasing the mutational load (APOBEC3B) and simultaneously decreasing AIRE expression, increased levels of neo-epitopes in non-AIRE regulated genes may have been generated which were potently immunogenic to CD8^+^ T cells (Fig. 4E). Overall, these data suggest that by re-setting the levels of AIRE, and/or introducing a higher mutational load, DMG tumours may become more amenable to T cell mediated immunotherapy. We are currently testing the hypothesis that AIRE routinely maintains the high levels of SELFNESS (lack of immunogenicity)^12^ of DMG tumours by having widespread access to multiple transcriptionally open genes (K27M mutant) allowing those tumour cells to be ‘ULTRA-SELF’ against which very few, if any reactive T cells exist (Figs. 4&5).

It was also possible to achieve the therapeutic effects of (increasing) AIRE expression in B16-F10 tumours by direct delivery with an AAV-AIRE expressing vector (Figs. 6A–D) through generation of heteroclitic T cell responses reactive against B16-F10 tumours (Fig. 6E). Therapy was dependent upon both CD4^+^ and CD8^+^ T cells (but not on NK cells) (Fig. 6F) and was significantly enhanced by combination with anti-PD-1 ICB (Fig. 6D). Although similar numbers of anti-B16-F10 CD8^+^ T cells were generated by AAV-8-AIRE and AAV-8-AIRE + anti-PD-1 ICB (by ELISPOT), the activity of those CD8^+^ T cells (amount of IFN-γ/CD8^+^ T cell) was significantly greater with addition of anti-PD-1 ICB (Fig. 6E) — showing the importance of de-repression of exhausted anti-tumour T cells by the anti-PD-1 treatment^22, 23, 24, 31^.

Mass spectrometry of peptides eluted from the MHC class I molecules of B16-F10, B16-F10-(shRNA-AIRE) and B16-F10-(AIRE) cells (Fig. 7A) showed that AIRE acts as a major controller of the total number of MHC-Class I-presented epitopes presented by tumour cells (Fig. 7B). By focusing on a single, well-defined SELF TAA TYRP1^51, 52, 66^, we showed that AIRE over-expression increased the total number of TYRP1 epitopes, whilst knock down of AIRE significantly decreased the number of TYRP1 epitopes (Fig. 7C). Three major classes of TYRP1 epitopes varied between the B16-F10, B16-F10-(shRNA-AIRE) and B16-F10(AIRE) cell lines — TYRP1 epitopes 1) shared between all three lines; 2) unique to one cell line; or 3) which shared a core sequence between B16-F10 parental and B16-F10.AIRE cells but which were 1–3 amino acids longer at the Carboxy terminal ends of the B16-F10-(AIRE)-eluted epitopes (Fig. 7G). In combination with anti-PD-1 ICB, both the Longer and the Unique peptide set significantly enhanced survival of B16-F10 tumour-bearing mice compared to controls (Fig. 7I) and primed recall responses against B16-F10 parental cells, with vaccination with the Longer peptides being significantly the most immunogenic (Fig. 7J). Figure 7K showed that CD4^+^ T cells from spleens of mice treated with DC/Longer + aPD-1 induced strong T helper IL-15 responses^62, 63, 64, 65^ *in vitro* from DC loaded with the shorter epitopes which were shared between B16-F10-(AIRE) and B16-F10 parental cells. CD4^+^ T cells from mice vaccinated with DC/Unique + aPD-1 were also able to activate weak IL-15 responses from DC loaded with the Unique peptides suggesting that these B16-(AIRE) expressed Unique epitopes generated in AIRE-over-expressing cells may contain T helper functions as well^62, 63, 64, 65^. The presence, and immunological potency, of these longer TYRP1 peptides in the B16-F10-(AIRE) expressing cells is consistent with reports that the immunogenicity of minimal length MHC Class I-binding peptides can be less than that of longer versions of the same peptides^68, 69, 70, 71, 72^ by allowing for better uptake and processing by professional APC and/or because long epitopes can contain epitopes for CD4^+^ T cells which enhance CD8^+^ T cell activation^68, 69^. Therefore, AIRE-mediated changes in epitope display by B16-F10 tumour cells led to the provision of CD4^+^ T cell helper epitopes capable of activating dendritic cells against both AIRE-specific and parental expressed epitopes of TAA such as TYRP1. Given the increased AIRE-induced immunogenicity of only these TYRP1 peptides (Figs. 7H&I), it seems likely that the combination of both higher levels of pre-existing epitopes, as well as generation of *de novo* CD8/helper CD4 epitopes, across a wide range of different potential AIRE-regulated TAA in the B16-F10 tumour cells will have contributed to the potent rejection responses following either AIRE dependent vaccination or *in vivo* delivery (Figs. 3C,5A,6D&F). Taken together, our data suggest that AIRE is acting not only as a master transcriptional regulator in tumour cells to increase or decrease the absolute numbers of (pre-existing) epitopes (Fig. 7B–D) but also to alter the quality of those epitopes — in particular by generating new (longer and unique) epitopes with the ability to provide CD4^+^ T cell help to CD8^+^ anti-tumour responses (Figs. 7G&K). We are currently investigating how AIRE — which contains a ubiquitin ligase domain^3^ — may affect the processing of antigens in the cell as well as their levels of their presentation.

RNAseq data from public databases^73, 74, 75^ shows that there is a significant decreased expression in melanoma compared to normal skin tissue (Supplemental Fig. 1A). These findings are consistent with our findings in Figs. 3B&C,6D that melanomas may evade immune clearance by reducing AIRE expression and that increasing AIRE in melanomas will induce greater immune recognition and tumour rejection. In addition, these data sets show a strong positive correlation between AIRE expression and markers of TCR signaling such as Zap70 and IFN-γ (Supplemental Fig. 1B) — again consistent with our studies showing that increased AIRE expression in melanomas induces strong indicators of T cell activation and breaking of tolerance (Figs. 2E&F, 3D and 6E). Finally, expression of AIRE is also predictive of response to immune therapies pancancer, p = 0.008 (Supplemental Fig. 1C) as with our findings that increasing AIRE expression in melanomas confers significant enhanced susceptibility to immune checkpoint blockade (Figs. 3C &6D). Taken together, these patient-derived data support the hypothesis that engineering increased AIRE expression in melanomas will enhance their rejection potential. However, our findings that decreasing levels of AIRE in DMG cells is associated with enhanced immunogenicity and tumour rejection indicate that the clinical efficacy associated with changes in AIRE levels (increased or decreased) will depend upon multiple factors such as tumour mutational load, neoantigen expression from AIRE-controlled vs non AIRE-controlled genes, and the use of AIRE as a biomarker between tumour, recurrence and normal tissue.

In summary, our data here are consistent with a model in which tumour cells express a level of AIRE thereby setting a profile of SELFNESS which cloaks the tumour cells from T cell attack allowing tumours to escape T cell clearance. Whereas mTEC in the thymus present SELFNESS profiles that encourage T cell recognition and activation/deletion, tumours employ a reverse mimicry of the mTEC by presenting epitopes selected for their lack of T cell recognition. Engineering changes in AIRE expression in tumour cells (either increasing or decreasing) alters this constellation of SELFNESS epitopes by changing the levels, and relative balance, of AIRE-regulated and non-AIRE-regulated pre-existing epitopes occupying the MHC Class I molecules. Changes in AIRE generate novel epitopes with potentially increased immunogenicity in the context of the T cell repertoire under which tumour T cell escape by AIRE expression was initially established. These novel epitopes can generate potent CD8^+^ T cell responses against themselves, but, most importantly, at least some subsets of those CD8^+^ T cells are heteroclitic and can recognize and kill parental tumours expressing the non-AIRE-modified epitopes. AIRE-mediated alterations in the quality of tumour associated epitopes can also support CD8^+^ T cell-mediated therapy by generating helper CD4^+^ T cell epitopes that are also cross reactive against tumour cells. Therefore, we propose that by re-setting the SELFNESS of tumour cells by altering levels of AIRE expression, it will be possible to generate both *de novo* effector CD8^+^ and helper CD4^+^ T cell responses which can recognize tumours without the need to identify specific TAA targets, which can be supported by additional immunotherapy interventions such as ICB, and which can lead to tumour clearance.

## MATERIALS AND METHODS

### Cell Lines

B16 murine melanoma cells were obtained from the ATCC prior to being modified with the relevant transgenes. Cell lines were authenticated by morphology, growth characteristics, PCR for melanoma specific gene expression (gp100, TYRP-1 and TYRP-2) and biologic behavior, tested mycoplasma-free and frozen. Cells were cultured for less than 3 months after thawing. The B16-OVA cell line was derived from a B16-F10 clone transfected with a pcDNA3.1ova plasmid obtained from Dr. Esteban Celis in 2000. B16-OVA cells were grown in DMEM (HyClone, Logan, UT, USA) + 10% FBS (Life Technologies) + 5 mg/mL G418 (Mediatech, Manassas, VA, USA) until challenge. B16-OVA-(AIRE), B16-F10-(AIRE), PKC-(AIRE), DIPG-XIII-(AIRE) and SOH-(AIRE) cells were generated by stable transduction of B16-OVA or B16-F10 cells with pCMV-Entry AIRE (Accession Number NM_009646) (Origene, Rockville, USA CAT#: MC218789). Cells were co-transfected with pBabePuro at a 10:1 ratio followed by selection in puromycin (1.25 μg/mL). B16-OVA-(shRNA-AIRE), B16-F10-(shRNA-AIRE), PKC-(shRNA-AIRE), DIPG-XIII-(shRNA-AIRE) and SOH-(shRNA-AIRE) cells were generated by transduction with shRNA lentiviral particles (4 unique 29mer target-specific shRNA) or with a scramble control (Origene, Rockville, USA Catalogue #TL510188V) followed by selection in puromycin (1.25 μg/mL). B16-TK cells were derived from a B16-F1 clone transfected with a plasmid expressing the HSV-1 TK gene in 1997/1998^56, 57, 58, 59^. Following stable selection in 1.25 μg/mL puromycin, these cells were shown to be sensitive to ganciclovir (Cymevene) at 5 μg/mL (19–21). The CT2A murine glioma cells were a gift from J. Sampson (Duke University).

The PKC cell line was derived from a genetically engineered mouse model that closely mirrors human DMG. This model makes use of an RCAS tumour virus system to induce PDGFβ and H3.3K27M overexpression in the context of p53 loss and is targeted to neonatal neural progenitor cells by the expression of the virus receptor under the control of the Nestin promoter^76, 77^. Briefly, brainstem tumours were established by implanting DF-1 producer cells transfected with the RCAS plasmids^76, 77^ into Nestin tv-a/p53 floxed mice, and the PKC cell line was established by explanting an established tumour from this model. K27M status of PKC was confirmed by sequence analysis.

DIPG-XIII and SOH are pediatric diffuse intrinsic pontine glioma (DIPG)/Diffuse Midline Glioma (DMG) cell lines were cultured in TSM media, which consists of 50% Neurobasal-A Medium, 50% DMEM/F-12, 10 mM HEPES solution, 1 mM MEM Sodium Pyruvate solution, 1× GlutaMAX Supplement, 1× Antibiotic/Antimycotic solution, 1× B-27 Supplement Minus Vitamin A, 20 ng/mL human epidermal growth factor (Shenandoah Biotech), 20 ng/mL human fibroblast growth factor basic-154 (Shenandoah Biotech), 10 ng/mL human PDGF-AA (Shenandoah Biotech), 10 ng/mL human PDGF-BB (Shenandoah Biotech), and 2 μg/mL heparin solution (StemCell Technologies).

Cells were tested for mycoplasma using the MycoAlert Mycoplasma Detection Kit (Lonza Rockland, Inc. ME, USA).

### Mice

6–8-week-old female C57BL/6 mice were purchased from Jackson Laboratories (Bar Harbor, Maine). The OT-I mouse strain is on a C57Bl/6 background and expresses a transgenic T-cell receptor Vα2/Vβ5 specific for the SIINFEKL peptide of ovalbumin in the context of MHC class I, H-2K^b^ as previously described and were bred at Mayo Clinic. The PMEL mouse strain is on a C57Bl/6 background and express a transgenic T-cell receptor Vα1/Vβ13 that recognizes amino acids 25–33 of gp100 presented by H2-D^b^ and were bred at Mayo Clinic.

### CD8^+^ T-cell preparation

For the preparation of naïve OT-I or PMEL T cells, spleen and lymph nodes from OT-I or PMEL transgenic mice were combined and crushed through a 100-μm filter to prepare a single cell suspension. RBC were removed by a 2-min incubation in ACK buffer (sterile distilled H_2_O containing 0.15 mol/L NH_4_Cl, 1.0 mmol/L KHCO_3_, and 0.1 mmol/L EDTA adjusted to pH 7.2–7.4). When indicated, CD8^+^ T cells were isolated using the MACS CD8α(Ly-2) Microbead magnetic cell sorting system (Miltenyi Biotec, Auburn, CA) and stained with CFSE dye (Molecular Probes, Invitrogen, Carlsbad, CA) according to the manufacturer’s instructions.

For preparation of activated OT-I T cells, single cell suspensions from spleen and lymph nodes were adjusted to 1.0 × 10^6^ cells/mL in Iscove’s modified Dulbecco’s medium plus 5% FCS, 10^− 5^ mol/L of 2-ME, 100 units/mL of penicillin, and 100 μg/mL of streptomycin and stimulated with 1 μg/mL of SIINFEKL peptide and 50 IU/mL of human interleukin 2 (Mayo Clinic Pharmacy). Every 2 to 3 days, one-half of the medium was removed and replaced with fresh medium containing 50 IU/mL of interleukin 2. For use *in vivo*, nonadherent and loosely adherent cells were harvested following one activation cycle of 3 to 5 days and viable cells were purified by density gradient centrifugation using Lympholyte-M (Cedarlane Laboratories) according to the manufacturer’s instructions. More than 90% of the cells expressed the Vα_2_ chain of the transgenic OT-I T cell receptor. CD8^+^ T cells were co-cultured with target tumour cells at various effector to target ratios as described in the text. Supernatants were assayed for IFN-γ by ELISA as directed in the manufacturer’s instructions (Mouse IFN-γ ELISA Kit, OptEIA, BD Biosciences, San Diego, CA).

### Viruses

VSV-GFP, VSV-hgp100 and VSV-ova were generated by cloning the appropriate cDNAs into the plasmid pVSV-XN2, as described in^78^. Monoclonal VSVs were obtained by plaque purification on BHK-21 cells. Concentration and purification were done by sucrose gradient centrifugation. Virus stock titers were measured by standard plaque assays of serially diluted samples on BHK-21 cells^60, 61, 78^.

### Generation of tumour experienced B16-TK (T.E.) CD8^+^ T cells

CD8^+^ T cells were prepared as described above from C57BL/6 mice that had been cured of subcutaneous B16-TK tumours following three weekly courses of GCV (50 mg/kg on days 5–9, 12–16, and 19–23). Cells were harvested between 60- and 80-days post tumour implantation.

### Quantitative RT-PCR and sequencing

RNA was prepared with the QIAGEN-RNeasy-MiniKit (Qiagen, Valencia, CA). 1 μg total RNA was reverse-transcribed in a 20 μl volume using oligo-(dT) primers using the First Strand cDNA Synthesis Kit (Roche, Indianapolis, IN). A cDNA equivalent of 1 ng RNA was amplified by PCR with gene-specific primers using GAPDH as loading control (mgapdh sense: TCATGACCACAGTCCATGCC; mgapdh antisense: TCAGCTCTGGGATGACCTTG.

Primers used to detect murine AIRE were: sense: 5’ atg gca ggt ggg gat gga atg c – 3’ and anti-sense: 5’ – GGA ACA CCT AGT CTG CGG GTG GA −3’ (NCBI Reference Sequence: NC_000076.7). CSDE1: Sense: 5′-ATG AGC TTT GAT CCA AAC CTTC − 3′; antisense: 5’-CAG TGT GTT TAT TGT TAT CAA TT −3’ (NCBI ReferenceSequence: NM_144901.4). TYRP2: Sense: 5’- GCAAGATTGCCTGTCTCTCCAG – 3’; antisense: 5’-CTTGAGAGTCCAGTGTTCCGTC-3’. (NCBI ReferenceSequence: NM_010024).

qRT-PCR was carried out using a LightCycler480 SYBRGreenI Master kit and a LightCycler480 instrument (Roche) according to the manufacturer’s instructions. The ΔΔC_T_ method was used to calculate the fold change in expression levels of target genes and GAPDH as an endogenous control for all treated samples relative to an untreated calibrator sample.

Levels of expression of the OVA transgene were assessed using the following primers:

Sense:ATGGGCTCCATCGGCGCAGCand antisense: CCGTCTACACAAAGGGGAATT and aligned to the reference sequence CAA23682.1.

### In vivo studies

All procedures were approved by the Mayo Foundation Institutional Animal Care and Use Committee. C57Bl/6 mice were purchased from The Jackson Laboratory (Bar Harbor, ME) at 6 to 8 weeks of age. To establish s.c. tumours, 5×10^5^ B16-OVA or B16-F10 cells in 100 μL of PBS were injected into the flank of mice. VSV-OVA, VSV-PMEL and VSV-GFP viral injections (100 μL) were done intra-venously at time points as described in each experiment (Fig. 3). AAV-8 injections were administered intra-tumourally (Fig. 6). Immune cell depletions were done by i.p. injections (0.1 mg/mouse) of anti-CD8 (Lyt 2.43) and anti-CD4 (GK1.5), both from the Monoclonal Antibody Core Facility, Mayo Clinic (Rochester, MN) and IgG control (ChromPure Rat IgG; Jackson ImmunoResearch, West Grove, PA) at day 4 after tumour implantation and then weekly thereafter. Fluorescence-activated cell sorting analysis of spleens and lymph nodes confirmed subset-specific depletions.

For immune checkpoint blockade, mice were treated intravenously or intra-peritoneally with anti-PD1 (0.25 mg; catalog no. BE0146; Bio X Cell), or isotype control rat IgG (catalog no. 012–000-003; Jackson ImmunoResearch) antibody at times described in each experiment.

For adoptive transfer experiments, mice were given naïve OT-I or PMEL T cells i.v. (10^7^ cells in 100 μL per injection) as described after tumour injection. For survival studies, tumour diameters were measured thrice weekly in two dimensions using calipers, and mice were sacrificed when tumour size was ∼1.0 × 1.0 cm in two perpendicular directions.

### ELISPOT and ELISA analysis for IFN-γ secretion

Spleens or tumour draining lymph nodes were removed from mice at the indicated times. For ELISA, a million cells were plated (unless otherwise indicated) in 24 well plates and incubated at 37°C with the indicated targets (peptides at 5 μg/mL *i.e.*, H-2K^b^-restricted peptides TRP-2_180− 188_SVYDFFVWL, ova SIINFEKL, synthesized at the Mayo Foundation Core facility) or cells as indicated. B16-OVA cells and variants were treated with 50 U of rIFN-γ for 24 hours prior to quantification of surface-expressed K2b/SIINFEKL or co-culture with T cells to increase the frequency of cells expressing MHC class I. Murine rIFN-γ (eBioscience, SD, USA, Cat# 14–8311-63). Cell-free supernatants were collected after 48 hours and tested by ELISA for IFN-γ (BD OptEIA^™^ Mouse IFN-γ ELISA Set; BD Biosciences Pharmingen, San Diego, CA, USA). For ELISPOT assays (Mouse Interferon-γ ELISpot^Plus^, MABTECH AB, Nacka Strand, Sweden), 1×10^5^ cells were plated into each well of a 96-well ELISPOT plate in triplicates and were re-stimulated for 48 hours at 37°C with the relevant targets (peptides or cells). Peptide-specific IFN-γ positive spots were detected according to the manufacturer’s protocol and were quantified by computer assisted image analyzer.

### Generation of murine bone marrow dendritic cells and vaccine preparation

Murine Bone Marrow Dendritic Cells were prepared from C57Bl/6 mice as described in^79, 80, 81, 82, 83^. Femurs were collected from C57/Bl6 mice, and bone marrow was flushed into RPMI media using a 25-gauge needle. Bone marrow was treated with Ammonium-Chloride-Potassium (ACK) Lysis Buffer, washed with serum-free RPMI, and then resuspended in RPMI supplemented with 10% FBS + 1× penicillin/streptomycin + 50 μM 2-Mercaptoethanol supplemented with murine granulocyte-macrophage colony-stimulating factor (GM-CSF (20 ng/mL; Peprotech). Cells were seeded at 10^6^ cells per well in 2 mL of a 24-well plate. Media were replaced with fresh murine GM-CSF-containing media on day 3. Bone marrow derived dendritic cells (BMDCs) were collected on day 5. B16-F10, B16-F10-(shRNA-AIRE) or B16-F10-(AIRE) tumour cells were expanded in T175 flasks. At 80–90% confluency, cells were trypsinized and washed three times in phosphate-buffered saline (PBS) (HyClone). Aliquots of 5×10^7^ cells were resuspended in a volume of 1 mL PBS and then freeze–thawed for three cycles in liquid nitrogen. Mature DC were then incubated with the tumour lysates at a ratio of 1 DC to 10 tumour cell equivalents at 37°C for 12 hours. Each vaccine comprised of 10^6^ mature DC loaded with the equivalent of 10^7^ tumour cells in 100 μL administered intravenously (i.v.) to mice.

### Murine T cell In Vitro Education and Restimulation

Splenocytes from naïve C57Bl/6 mice were co-cultured with live PKC, PKC(shRNA-AIRE) or PKC-(CMV-AIRE) cells, pre-treated for 24 hours with IFN-γ to enhance MHC Class I presentation, at a ratio of 10:1 for three days with IL-2. On days 6/7 and 9/10, co-cultures were re-plenished with live, IFN-γ -pre-treated PKC variant tumour cells. After 2 weeks of culture, CD8^+^ T cells were recovered by magnetic bead isolation and co-cultured with 10^5^ parental PKC tumour cells at a ratio of between 5:1 and 10:1. Cell-free supernatants were collected after 48 hours and tested by ELISA for IFN-γ (BD OptEIA^™^ Mouse IFN-γ ELISA Set; BD Biosciences Pharmingen, San Diego, CA, USA).

### APOBEC3 overexpression

Human DMG DIPG-XIII or SOH cell lines, or their AIRE over-expressing or knocked down variants, were infected with a retroviral vector encoding either full length functional APOBEC3B (APOBEC3B^ACTIVE^) or a mutated, catalytically inactive form of APOBEC3B (APOBEC3B^INACTIVE^) as a negative control. Forty-eight hours post transduction with either pBABE-Hygro APOBEC3B^ACTIVE^ or pBABE-Hygro APOBEC3B^INACTIVE^ viruses, bulk populations of cells were selected in hygromycin for no more than 2 weeks and used for experiments. Overexpression of APOBEC3B was confirmed by both Western Blot (using a rabbit monoclonal anti-human APOBEC3B (184990, Abcam, San Francisco, CA)) and qrtPCR as previously described^42^. Over-expression of APOBEC3B is toxic because mutagenesis by APOBEC3B is tolerable to the cell up to a certain threshold, APOBEC3B cells were used within 14 days to prevent the accumulation of toxic mutations killing the cells (more details in^42^).

### Human T cell In Vitro Education and Restimulation.

Fresh PBMCs from a healthy donor were acquired from the Mayo Clinic Blood Bank. CD3^+^ T cells were isolated using a magnetic sorting kit (Miltenyi Biotech) and activated using CD3/CD28 beads (ThermoFisher). T cells were immediately co-cultured at a ratio of 10:1 with autologous dendritic cells loaded with DMG cell line lysates.

Autologous monocyte-derived dendritic cells were matured by isolating CD14^+^ cells by magnetic sorting (Miltenyi Biotech), followed by incubation with human GM-CSF (800 U/mL) and IL-4 (1000 U/mL). On Days 3 and 5, media was replaced with human GM-CSF (1600 U/mL) and IL-4 (1000 U/mL). On Day 7, non-adherent cells were collected, washed with PBS, and resuspended in medium containing GM-CSF (800 U/mL), IL-4 (1000 U/mL), TNF-alpha (1100 U/mL), IL-1beta (1870 U/mL), IL-6 (1000 U/mL), and PGE2 (1 μg/mL). On each of the first three days of co-culture, cell lysates of DMG, DMG-(shRNA-AIRE) or DMG-(CMV-AIRE) were added to the culture at an approximate ratio of DMG cell (lysate):DC of 10:1. Two days later, dendritic cells were harvested for co-incubation with activated T cells at a ratio of 1:10.

Ten days after initial DC(Lysate)/CD8^+^ T cell co-culture, CD8^+^ T cells were re-isolated using magnetic bead sorting (Miltenyi Biotech), and then co-cultured with interferon gamma pre-treated (200U/mL for 12 hours) parental DMG cells for 72 hours, followed by interferon gamma ELISPOT (R&D).

### Flow cytometry

For analysis of phenotype, 1×10^6^ cells were washed in 1X PBS containing 0.1% BSA and 0.01% sodium azide (FACS buffer), re-suspended in 50 μl of FACS buffer, and exposed to fluorochrome-conjugated primary antibodies for 30 min at 4°C. The mouse IgG125-D1.16 antibody is specific for the MHC class I molecule Kb bound to the peptide SIINFEKL (Kb-SIINFEKL) (https://www.biolegend.com/en-us/search-results/pe-anti-mouse-h-2kb-bound-to-siinfekl-antibody-7247?gclid=EAIaIQobChMIm8fyo8XThgMVvCrUAR0D9Q9KEAAYAyAAEgJ1HvD_BwE (Biolegend San Diego, USA). Cells were then washed and resuspended in 500 μl of PBS containing 4% formaldehyde.^10^ Cells were subjected to flow cytometry and data were analyzed using CellQuest software (BD Biosciences, San Jose, CA, USA) or FlowJo (Tree Star, Inc., Ashland, OR, USA).

### MHC Immunoprecipitation and mass spectrometry

MHC class I immunoprecipitation from B16-F10 cells was performed as previously described using the anti-H-2K^b^ antibody (Clone Y-3)^84^. Briefly, B16-F10 cells were propagated to 1×10^9^ total cells in 50, 150 cm dishes. Cells were trypsinized and collected to obtain a cell suspension. Cells were washed two times in PBS, pelleted, and then flash frozen in liquid nitrogen and stored at −80°C until prepared for immunoprecipitation. Immunoprecipitation columns (BioRad) were prepared with 4 mL Protein A Sepharose resin (CaptivA PriMAB) crosslinked with 6 mg anti-H-2K^B^ (Clone Y-3, BioXCell). Cell pellets were lysed with 20 mL 0.5% IPEGAL lysis buffer with 2X protease inhibitors (Roche, EDTA free). Lysates were centrifuged at 2,000g for 10 minutes. Supernatant was collected and ultracentrifuged at 100,000g for 75 minutes. Supernatant was collected and filtered through a 0.45 μm filter. Lysates were precleared on columns with 2 mL sephrose A resin and then loaded onto antibody bound columns. Lysates were allowed to flow through by gravity and then washed with 100 mL of wash buffer 1 (0.005% IPEGAL, 50mM Tris, pH 8, 150 mM NaCl, 5 mM EDTA, 100 μM PMSF, 1 μg/mL pepstatin A), wash buffer 2 (50 mM Tris, pH 8, 150 mM NaCl), 3 (50 mM Tris, pH 8, 450 mM NaCl), and wash buffer 4 (50 mM Tris, pH 8). Bound MHC complexes were eluted in 10%v/v acetic acid and sent for LC-MS/MS at the Mayo Clinic Proteomics Core.

### Statistics

Survival curves were analyzed by the Log-Rank test. Student’s T tests, one-way ANOVA and two-way ANOVA were applied for *in vitro* assays as appropriate. Statistical significance was set at *p* < 0.05 for all experiments.

## Supplementary Material

Supplement 1
