# Supplementary material for: Cancer Immunotherapy Using AIRE Conditioning of the Tumor Epitopeome
Source: Res Sq. 2024 Nov 15:rs.3.rs-5411393. Preprint. [Version 1] doi: 10.21203/rs.3.rs-5411393/v1 (PMC11601838; doi:10.21203/rs.3.rs-5411393/v1)
Supplement: Supplement 1 [file NIHPPRS5411393V1-supplement-1.pdf]

Supplementary Files

This is a list of supplementary files associated with this preprint. Click to download.

- SupplementalFigure1.docx
